# Intertidal exposure favors the soft-studded armor of adaptive mussel coatings

**DOI:** 10.1038/s41467-018-05952-5

**Published:** 2018-08-24

**Authors:** Christophe A. Monnier, Daniel G. DeMartini, J. Herbert Waite

**Affiliations:** 1 Marine Science Institute, University of California, Santa Barbara, CA 93106 USA; 2 Department of Molecular, Cellular and Developmental Biology, University of California, Santa Barbara, CA 93106 USA

## Abstract

The mussel cuticle, a thin layer that shields byssal threads from environmental exposure, is a model among high-performance coatings for being both hard and hyper-extensible. However, despite avid interest in translating its features into an engineered material, the mechanisms underlying this performance are manifold and incompletely understood. To deepen our understanding of this biomaterial, we explore here the ultrastructural, scratch-resistant, and mechanical features at the submicrometer scale and relate our observations to individual cuticular components. These investigations show that cuticle nanomechanics are governed by granular microinclusions/nanoinclusions, which, contrary to previous interpretations, are three-fold softer than the surrounding matrix. This adaptation, which is found across several related mussel species, is linked to the level of hydration and presumed to maintain bulk performance during tidal exposures. Given the interest in implementing transfer of biological principles to modern materials, these findings may have noteworthy implications for the design of durable synthetic coatings.

## Abstract (web summary)

There is interest in the development of mussel inspired materials; however, this requires an understanding of the materials. Here, the authors report on an investigation into the properties of mussel cuticle from different species that challenges conventional wisdom about particle filled composites.

## Introduction

Functional coatings, though often inconspicuous and deceptively simple in appearance, are essential in providing the underlying bulk material with durability against physical and chemical stresses. However, the inherent inverse relationship between stiffness and extensibility frequently impedes the design of man-made versions and usually results in the promotion of one of these features at the expense of the other^1^. Biological counterparts on the other hand are known to sidestep this trade-off via remarkable structural and biochemical arrangements^2^. A prime example is the mussel cuticle: this non-mineralized coating^3^, which shields the byssal threads from degradation in both physically and chemically aggressive environments, is capable of extending to strains of up to 120%^4–6^ despite being as stiff as an epoxy resin^7^. This unusual blend, without a counterpart among synthetic formulations, makes this coating a formidable line of defense against the relentless challenges encountered in littoral habitats^8^ and has led to its appeal as a paradigm for designing tough and durable materials^9,10^.

The functional advantages of the cuticle have been correlated with its microscopic architecture, which has been described as a dual-phase system of both submicrometer inclusions (a.k.a. granules, presumably made of condensed mussel foot protein 1)^11,12^ and an amorphous matrix. Previously highlighted by Holten-Andersen et al., this arrangement was shown to correlate with the need for energy dissipation^13^, whereas Harrington et al. demonstrated via Raman spectroscopy that dynamic self-healing metal–catechol complexes^14^ are concentrated in the granules^15^. As a result, these granules were deduced to be rigid fillers within the apparently soft and extensible matrix and therefore credited for toughening the cuticle in a fashion comparable to that seen in particle-reinforced composites (i.e., by acting to reduce crack propagation and abrasion^13,16^). However, while this view is consistent with modern concepts of multiphase wear-resistant materials, whether it explains in situ performance has not been demonstrated. Moreover, specific questions arise from this scheme, such as why damage should be deflected around highly regenerative domains (for which metal–catechol bonds are especially well-adapted)^17^ or as to how structural variations in the granular phase influence functional and mechanical responses. To date, these subjects have remained unexplored, presumably due to the difficulty of discerning the specific phases with the analytical methods currently available, and imply that some cuticle features may still be unknown or elusive.

Here we examine the cuticles of four mussel species with different granular morphologies to better understand the material properties of these coatings and explore the correlation between architecture and wear. In situ atomic force microscopy (AFM) is used to identify the nanometer-scale scratch and indentation resistance, while transmission electron microscopy (TEM) images and tomograms provide precise structural information of the cuticles and their precursors. From these analyses, an unexpected discovery is emerging: the granules, not the surrounding matrix, are the softer components of the cuticle. Moreover, they are more hygroscopic than the matrix, suggesting their main function to be akin to that of plasticizers and hydration reservoirs rather than reducing abrasion. While these findings invert our view on cuticle mechanics, they highlight the roles and contributions of the individual phases to overall material performance and offer alternative explanations on how this tough biological coating remains durable in harsh ambient environments.

## Results

### Habitats and ultrastructural characteristics

Coastal habitats are turbulent environments with respect to a variety of fluctuating physicochemical and ecological challenges (e.g. tidal exposure/desiccation, intense wave action, abrasion, large swings in temperature and salinity, predation, etc.)^4,18^. Despite such instability, many organisms have evolved special adaptations to occupy these habitats. Mussels are exemplary in this respect and thrive along wave-battered coastlines. This is made possible by the byssus, a bundle of threads endowed with highly specialized and exceptional material properties to ensure their survival^19^.

**Fig. 1:**
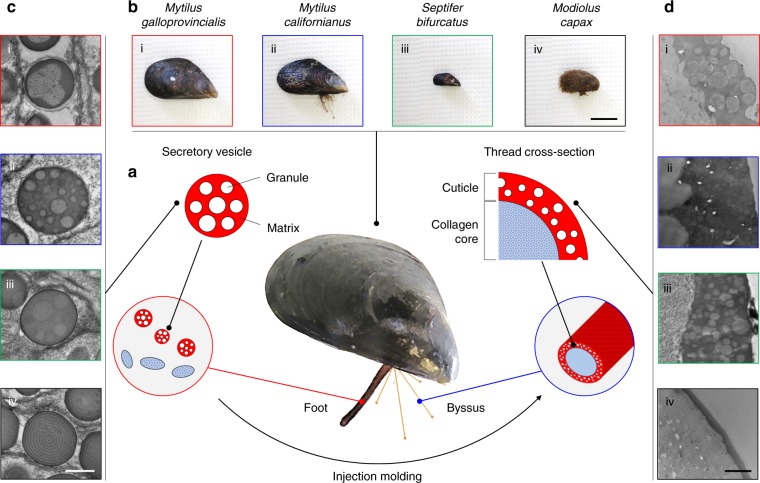
Fabrication, structural diversity and individual components of the mussel cuticle. All panels labeled with (i) represent *M. galloprovincialis*, (ii) *M. californianus*, (iii) *S. bifurcatus*, and (iv) *M. capax*. **a** Scheme of cuticle processing in the mussel highlighting the prefabrication of individual granules and surrounding matrix within secretory vesicles in the foot and via injection molding, leading finally to a coalescence and hardening of myriad secreted vesicles as the byssal cuticle; **b** Images of the four types of mussels selected for this study, (scale bar = 2 cm). **c** TEM micrographs of the cuticle precursors (scale bar = 0.5 µm); **d** TEM micrographs of lateral cross-sections of mussel threads (scale bar = 1 µm)

Each byssal thread is fabricated in minutes by a process resembling synthetic polymer injection molding^20–22^ and consists of a collagenous core coated by a thin toughened cuticle (Fig. 1a). This cuticle shares the tensile attributes of the core while protecting it against environmental impacts and is micro-adapted to the locally experienced challenges. Thus, to cover eventual structural varieties across species and niches, we selected four related Mytilids (Supplementary Figure 1) from the Southern Californian coastline as model organisms for our investigations, i.e., *Mytilus galloprovincialis* (Lamarck, 1819)^23^, *Mytilus californianus* (Conrad, 1837)^23^, *Septifer bifurcatus* (Conrad, 1837)^23^, and *Modiolus capax* (Conrad, 1837)^23^ (Fig. 1b, i–iv). Indeed, microscopic differences between their cuticular architectures are apparent in TEM images of the secretory vesicles (i.e., the cuticle precursors in the foot, Fig. 1c)^21^ and cuticles (Fig. 1d). However, distinct similarities are apparent as well: *M. galloprovincialis*, *M. californianus*, and *S. bifurcatus*, whose cuticles contain granular structures of varying complexity (Fig. 1c, d, i–iii), are all residents of the intertidal zone: the two former, which share comparable spherical granules, are found in exposed wave-battered niches, whereas the latter with its large, sponge-like granules prefers more protected spaces. Conversely, *M. capax*, which stands out with a featureless cuticle, is found in deeper subtidal waters, where it is usually sheltered by sediment, kelp holdfasts, or mussel clusters (Fig. 1d, iv). These observations support interpretations by Holten-Andersen et al. that the granules, whose occurrence has been linked to the exceptional cuticle properties, are typically expressed in high-energy zones and omitted in less turbulent ones^13^. Another noteworthy relation is that all phase-separated cuticles undergo periodic dehydration and rehydration cycles because of tidal ebb and flow. However, whether such cycling is correlated with this distinct macroscopic organization has not been explored, nor if the microstructural differences seen across species contribute to specific cuticle performance features, e.g. abrasion resistance.

### Resistance to scratches

**Fig. 2:**
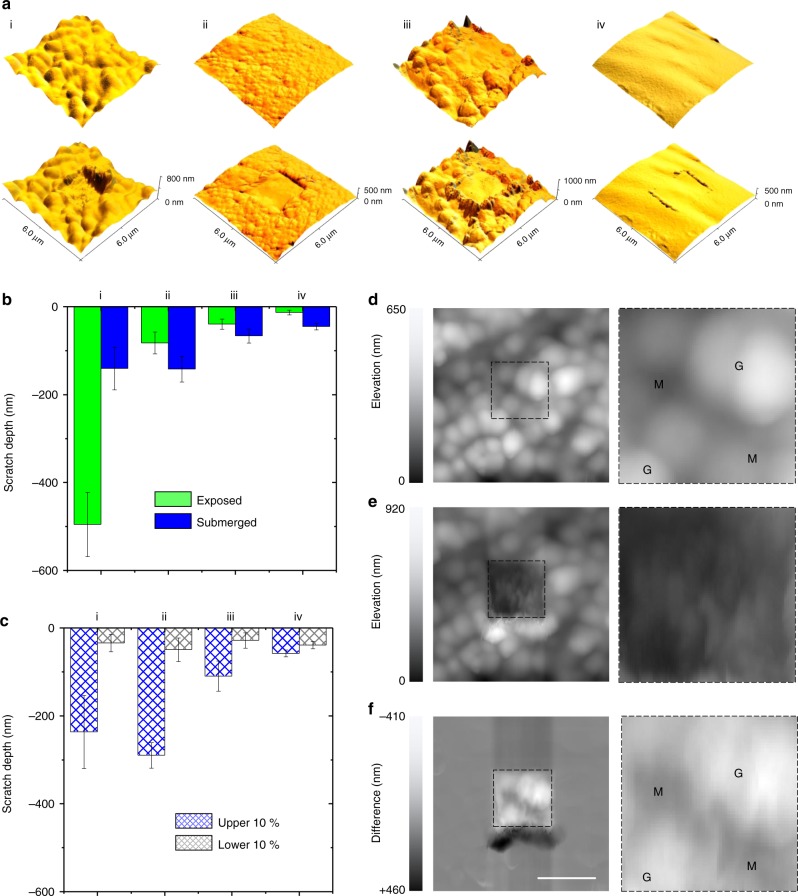
2D AFM scratch tests on the cuticle surfaces. All panels labeled with (i) represent *M. galloprovincialis*, (ii) *M. californianus*, (iii) *S. bifurcatus*, and (iv) *M. capax*. **a** Representative AFM topographic images of all mussel thread surfaces without (top) and with (bottom) scratch traces. **b** Scratch depths as a function of species and hydration. **c** The hydrated datasets are further partitioned in the upper and lower 10%. **d**–**f** A representative scratch test on a *M. galloprovincialis* thread (scale bar = 2 µm), with AFM height channel recordings prior (**d**) and after (**e**) damaging the surface, as well as a difference image of both (**f**). Zoom-ins of the region of interest are included on the right (M = matrix and G = granules) (Bars are mean ± s.e.m., *n* = four biological replicates)

To investigate these possibilities, we first explored how these four cuticles hold up against aggressive physical action. To do so, AFM^24^-based two-dimensional scratch tests^25–27^ (Supplementary Figure 2), for which the tip is used as an abrasive utensil, were conducted on both submerged and exposed (i.e., a situation comparable to that experienced during low tide) threads to faithfully reflect actual performance conditions. All parameters for these investigations (i.e., applied force and number of cycles) were carefully optimized and set to outweigh topographic contributions (Supplementary Figure 3, Supplementary Figure 4). Building upon previous cuticle concepts (i.e., of hard spheres embedded in a soft matrix), an anticipated outcome was that scratch resistance would be high within granule-containing cuticles but lower in deficient ones. Instead, opposite trends were observed: substantial damage rates were sustained by the cuticles of all intertidal species (Fig. 2a, i–iii), compared with nominal damage on that of *M. capax* (Fig. 2a, iv). Similar, yet slightly deeper scratch depths were reached on fully submerged threads (Fig. 2b), with the only exception being those of *M. galloprovincialis* (i.e., with greatly reduced, yet still significant damage rates in both states). Sorting data between extremes (i.e., the lower and upper 10% of the scratch depths, Fig. 2c) showed that the lower damage rates range from 30 to 50 nm across all species, whereas the upper ends fluctuate significantly between 100 and 300 nm (with exception of the granule-deficient cuticle of *M. capax*). This variability indicates that one of the two phases is more prone to damage than the other. A closer look at a representative scratch test on a cuticle of *M. galloprovincialis* (Fig. 2d–f) reveals which one it is: the granules, which are clearly discernible in the initial topographic image despite being beneath the surface (Fig. 2d), are being eroded, hollowed out, and/or shredded more readily than the matrix. This tendency is especially evident in the difference image (Fig. 2f). As wear behavior is closely coupled with the local mechanical properties, these results show that the granules do not endow a composite cuticle with better shielding against abrasion than a granule-deficient one.

### Nanomechanical mismatches and the effects of hydration

**Fig. 3:**
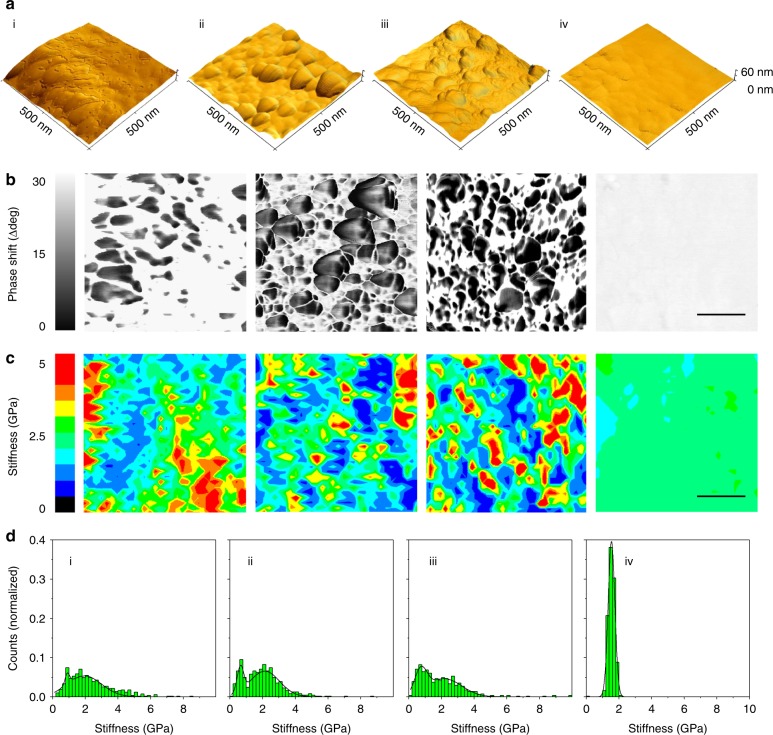
Nanomechanical investigations of the cuticle granules. All panels labeled with (i) represent *M. galloprovincialis*, (ii) *M. californianus*, (iii) *S. bifurcatus*, and (iv) *M. capax*. **a** High-resolution AFM topographical renderings of the abraded, exposed cuticles. **b** Phase shift images of AFM renderings in **a** (scale bar = 150 nm). **c** The granular structures in this channel are discernible by phase shifts to lower degrees. Corresponding color-coded force spectroscopy maps (32 × 32 pixels, scale bar = 150 nm) across these regions show a distinct link between the nanomechanical behavior and phase shifts. **d** Summary of binned and fitted stiffness values

**Fig. 4:**
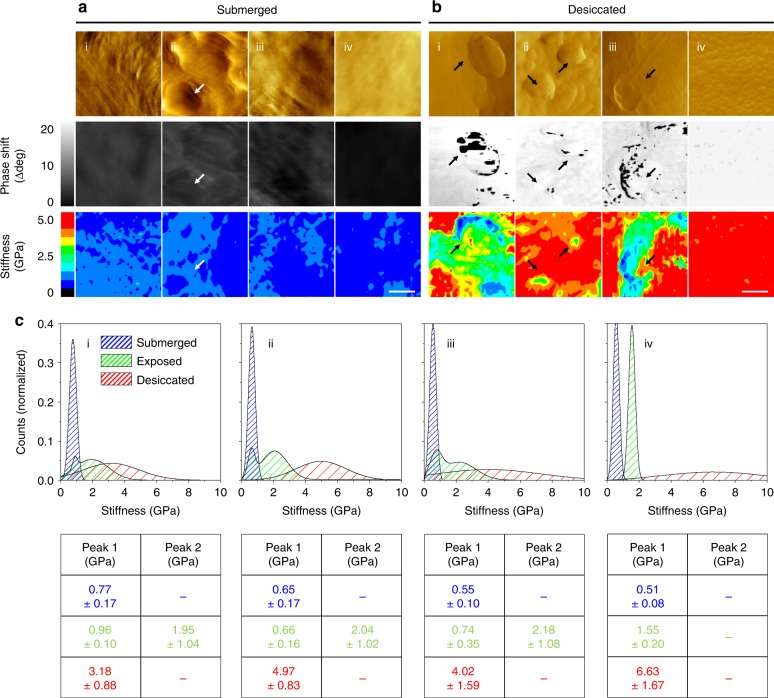
Hydration effects on cuticle nanomechanics. All panels labeled with (i) represent *M. galloprovincialis*, (ii) *M. californianus*, (iii) *S. bifurcatus*, and (iv) *M. capax*. **a**, **b** AFM amplitude (top row), phase (middle row), and force spectroscopic (lower row, 32 x 32 pixels) images of the submerged (**a**) and desiccated (**b**) cuticles of all species (scale bar = 150 nm). Arrows indicate the granular structures within the individual channels. **c** Corresponding fitted stiffness distributions of all force maps (submerged/blue, exposed/green, and desiccated/red, see Supplementary Figure 6 for unfitted raw datasets), along with the peak values (*n* = 576 binned datapoints, mean ± s.d.), are overlaid and summarized

These unexpected results provoked us to take a step back and scrutinize the nanomechanical properties of the individual cuticle components. As seen in AFM images recorded within the abraded areas, multiple granular structures, which are otherwise covered by matrix material (Fig. 1d), are clearly distinguishable (especially in *M. californianus*, Fig. 3a, Supplementary Figure 2d) and apparently intact (Supplementary Figure 5). More comprehensive investigations were thus carried out on these uncovered granules despite possible alterations of their properties through the scratch tests. In doing so however, potential artifacts arising from microscale sectioning treatments (e.g., embedding, chemical fixation, freezing, or slicing), which are even more likely to perturb overall structure and biomechanics than scratch tests, were avoided. AFM phase imaging, which takes advantage of viscoelastic differences to generate contrast over topographic features^28^, highlights the granules particularly well (Fig. 3b, Supplementary Figure 5). Although deducing the exact reason for these variations from AFM alone is risky, the featureless cuticle of *M. capax*, which can be loosely regarded as a granule-free control, offers a hint in this respect: its homogenous appearance confirms that large phase shifts, as seen in all dual-phase cuticles, are associated with the granules. Force spectroscopic maps recorded over these same regions look into the nature of this mismatch and reveal a variety of both soft and hard phenotypes (Fig. 3c). When compared with the corresponding phase images (Fig. 3b), it is evident that the compliant elements are better correlated with the granules than the matrix (these areas, as well as the entire cuticle of *M. capax*, appear rather rigid instead). This mechanical depiction is further evident in the stiffness profiles (Fig. 3d): histogram plots of the recorded values exhibit a unimodal distribution for *M. capax*, whereas bimodal distributions are observed in *M. galloprovincialis*, *M. californianus*, and *S. bifurcatus*. As deduced from the force spectroscopic maps, these two peaks represent the two cuticular phases, the softer one (i.e., between 0.66 and 0.96 GPa) being that of the granules and the stiffer one (i.e., between 1.95 and 2.18 GPa) that of the matrix. Measurements conducted on submerged threads, however, show another trend: when fully hydrated, stiffness distributions become homogeneous in the cuticles of all species (i.e., between 0.51 and 0.77 GPa), and neither phase is clearly discernible anymore (Fig. 4a). Occasionally evident, the granules, however, still appear marginally softer (as seen in *M. californianus*, Fig. 4a, ii, marked with arrows). Conversely, a total deterioration of mechanical uniformity is observed when moisture is artificially removed. This is reflected by a dramatic increase of stiffness and spread when the cuticle is freeze-dried (i.e., up to 6.63 GPa, Fig. 4b) and implies a loss of function of the material.

Relating these biomechanical profiles to one another (Fig. 4c) identifies the role and effects of water on the granules and reveals distinct hydration behavior of the individual cuticle components. Although elevated water contents were expected to reduce stiffness (and vice versa)^29,30^, the peaks of the hydrated cuticles coincide closely with the soft granular peaks expressed at intermediate hydration levels (Fig. 4c). This indicates that the granules retain mechanical compliance and hydration during emersion, whereas the matrix dries out and stiffens more rapidly. In doing so, cuticle brittleness and loss of function may be delayed, supporting the concept that the granules serve as water reservoirs and macromolecular plasticizers during exposure at low tide.

### Tomographic renderings and volume fractions

**Fig. 5:**
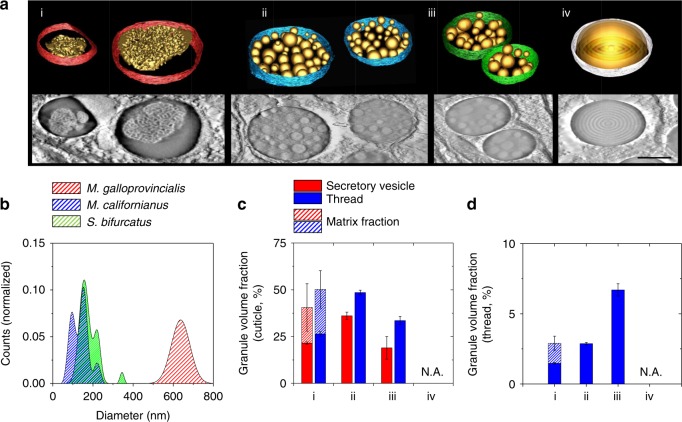
3D structural features and volume fractions. All panels labeled with (i) represent *M. galloprovincialis*, (ii) *M. californianus*, (iii) *S. bifurcatus*, and (iv) *M. capax*. **a** Reconstructed tomographic representations of the secretory vesicles and encapsulated structures (scale bar = 500 nm); **b** fitted size distributions of *M. galloprovincialis* (red, *n* = 122), *M. californianus* (blue, *n* = 176), and *S. bifurcatus* (green, *n* = 102) as deduced from TEM and electron tomographic investigations. **c** Granular volume fractions from the secretory vesicles (blue) compared to those of the cuticle (green, see Supplementary Figure 7 for full datasets), as well as normalized to the total thread size (**d**), show an increase in granular density after thread formation. Note that the intergranular matrix space in *M. galloprovincialis* is taken into account as well (dazzled segments, *n* = four tomographic sections, technical replicates, 1 µm × 1 µm × 60 *Z* slices, mean ± s.e.m.)

The apparent relationship between scratch resistance and mechanical investigations encouraged us to reinspect the cuticle architectures in deeper detail. Given that ultrastructural properties are often obscured or incompletely depicted in two-dimensional images, we reverted to electron tomography to acquire more precise representations and accurate volume fractions. Three-dimensional renderings of the secretory vesicles (Fig. 5a) highlight once more the granular varieties across the different species at an even greater detail. The perforated granules of *M. galloprovincialis*, for example (Fig. 5a, left), consist of approximately 50% of matrix volume, whereas the more subtle and compact morphologies of *M. californianus* and *S. bifurcatus* exhibit partially overlapping size distributions and gradual spacing between peaks (i.e., of >60 nm). Interestingly, the cuticle *M. californianus* exhibits the smallest granules of all (i.e., >95 nm, Fig. 5b, Supplementary Figure 7). Indeed, this discrete difference is reflected in its high volume fraction of >49% (Fig. 5c), which exceeds its counterparts provided that the intergranular matrix volume of *M. galloprovincialis* is subtracted (i.e., 50.2% versus 26.4%). More importantly, however, these volume fractions, if assuming that porous structures are less robust against abrasive action than solid spheres, appear correlated with the scratch tests, as the cuticles with high matrix content (i.e., *S. bifurcatus* with >34% and *M. capax* with 0%, respectively) sequentially exhibit shallower scratch depths.

While the volume fractions put the compositional contributions into context, they divulge a compelling clue on cuticle formation: it appears that the matrix condenses during the transition from the secretory vesicles to the thread, as an increase of 20% is reproducibly observed between the two (Fig. 5c). On the other hand, the granular structures, which are preassembled before secretion, appear unaltered by this process. This behavior, which is similar to that seen in standard covalent-crosslinking thermosets, shows that cuticle assembly bears strong similarities to a solvent-free, Type III self-stratified coating^31–33^, in that one substance initially phase-separates and the other subsequently cures (i.e., prior condensation of the granules in the foot, and matrix crosslinking during the molding process). Moreover, this explains the rapid processing time and lack of contamination between layers and further clarifies why an elevated matrix stiffness vis-à-vis the granules is observed, as stiff (i.e., cured) covalent crosslinks would predominate in the former versus dynamic metal-catechol complexes in the latter.

Finally, normalizing these fractions to total thread volume (Supplementary Figure 8) yields a concluding hint about granule function, as these values appear correlated with tidal exposure (Fig. 5d). *S. bifurcatus*, which colonizes similar (though frequently more super-littoral) niches as *M. californianus*, is arguably the most exposed of all species and exhibits the highest granule volume per thread (i.e., >7%). *M. galloprovincialis*, on the other hand, which prefers more sheltered habitats (i.e., bays, docks, and coves and is often nestled beneath *M. californianus*), is on the lower end of this range (i.e., <1.5%). In combination with all previously presented data, the featureless and permanently submerged cuticle of *M. capax* underscores once more the granules as key components in preserving material performance during wet and dry cycles.

## Discussion

Using different approaches, we have shown at several levels that the nanomechanical relationship between cuticle matrix and granules is opposite to that espoused in earlier models and that the role of the granules is more complex than previously assumed. First, they do not stiffen the cuticle but are instead responsible for keeping it plasticized via hydration. Second, granule hydration and plasticity, in contrast to the surrounding matrix, persist during emersion, preventing the cuticle from rapidly becoming stiff and brittle. Therein, although it stands to reason that these elastic fillers are safety nets implemented to preserve flexibility during periods of low tide, the main advantage of this arrangement would be that they, not the matrix, carry the bulk of deformation energy sustained during tension, as is seen in rubber-toughened epoxies^34,35^. Though counterintuitive at first, this concept is plausible when pondering granule composition: as metal–catechol interactions rapidly self-heal when broken^17,36,37^ and increase tensile toughness in polymer networks^38^, it is conceivable that load and damage should be localized to the granules, where their density is highest^15^. The preserved hydrated conditions in the granules would further ensure that regenerative and dynamic processes are continuously active, eventually ensuring that the coating remains functional and durable in and out of water.

Although the mussel cuticle resembles cutting-edge synthetic- coatings, its performance, processing, and solvent-free fabrication are still unrivaled. Given these, better comprehending its underlying assembly and functional mechanisms has fundamental scientific and technological value, especially in the development of structures with efficient energy-dissipative, protective, and self-healing properties. Consequently, these implications will hopefully benefit the design of bio-based coatings and support the increasing trends of moving away from petroleum-based, layered materials toward sustainable versions.

## Methods

### Organisms

All organisms used in this study were collected from the surrounding Santa Barbara coastline and allowed to reattach to Plexiglas surfaces within an open circulating seawater tank. Fresh threads of <24 h of age were then used for all experiments and kept in saltwater until being investigated. Other threads were dehydrated by lyophilization overnight.

### AFM (sample preparation and imaging)

All experiments were conducted on a MFP-3D atomic force microscope (Asylum Research, Santa Barbara, USA) mounted on an inverted light microscope. AppNano FORTA silicon tips were used for all experiments, and their exact spring constant values (which usually ranged between 2 and 3.6 nN/nm) were experimentally assessed prior to every usage by the thermal tune method. Deflection sensitivity was determined using glass as an indefinitely stiff reference material. Sample preparation involved cautiously mounting freshly collected threads on glass slides with standard two-component glue without undergoing any kind of treatment. Minimal amounts of adhesives were used to avoid misleading measurements, and mechanical manipulations during sample preparations were kept minimal at all times. Threads investigated in seawater were left to incubate for 30 min after being mounted under the AFM, leaving the cantilever enough time to equilibrate to the surrounding liquid. Exposed threads were investigated between 1 and 2 h after being removed from saltwater. All images were recorded in AC mode at scan rates of 0.7 Hz.

### Scratch tests

Comparative scratch tests were conducted based on the topographical difference method^27^. Cuticle surfaces were abraded by imaging in contact mode with an excessive applied load of 1 µN at a scan rate of 2 Hz. For this, an even region of interest of 2 × 2 µm (128 lines) was selected and repetitively scratched over ten cycles. Topographic profiles collected prior to and after scratching were then overlaid to evaluate how much material was displaced. Overall scratch depths were assessed by averaging all depth points over the given region of interest.

### Force spectroscopy

All force spectroscopic measurements were performed with a loading force of 75 nN and loading/unloading rate of 250 nm/s. The loading force was chosen with respect to Buckle’s one-tenth law^39^ to mitigate any influence of the underlying layers. High-resolution force spectroscopic maps of 32 × 32 pixels (1024 force curves) over a square of 500 nm were recorded over a previously imaged surface. Lower-resolution maps (24 × 24 pixels, 576 force curves) over the same region were subsequently registered for statistical analysis. Stiffness values were extracted with the Asylum Research MFP-3D Hertz analysis tool by using the upper 60% of the approach curve, a half-angle of 20°, and a Poisson ratio of 0.33 to fit the model. The calculated stiffness values were then spatially plotted to yield a color-coded stiffness map with OriginLabs 8.5. A two-dimensional spline interpolation was performed on these maps to smoothen the visual presentation of the data. Peak values in the binned stiffness distributions were identified via the Origin Peak Analysis feature.

### TEM (sample preparation and tomography)

Mussel feet and threads were prepared for electron microscopy by optimizing the fixation conditions. All fixation and washing steps were performed on ice. Fresh tissues were fixed in 2% formaldehyde, 2.5% glutaraldehyde, and fixation buffer (200 mM sodium cacodylate, 300 mM NaCl, pH 7.2) for 2 h. The samples were washed three times (10 min each) in degassed fixation buffer and then post-fixed in 2% osmium tetroxide in degassed fixation buffer for 2 h. The samples were then washed 4 times (10 min each) in degassed deionized water and then dehydrated through a graded series of ethanol washes (25, 50, 75, 90, 100, 100, 100% ethanol, 10 min each). Solvent was then switched to propylene oxide by washing in 33, 66, 100, 100, 100% propylene oxide in ethanol. The samples were then infiltrated with epoxy resin (Embed812, Electron Microscopy Sciences, Hatfield, USA) incubating the sample in resin diluted in propylene oxide as follows: 33% (2 h), 66% (16 h), and 100% (4 h). Finally, samples were placed in molds and cured at 60 °C for 24 h. Thin sections (60–80 nm) for TEM and semithin sections (300 nm) for electron tomography were cut on a EM UC6 ultramicrotome (Leica Biosystems). Sections were mounted on copper TEM grids and post-stained on drops of uranyl acetate and lead citrate following standard protocols^40^.

All samples were investigated with a Tecnai G2 transmission electron microscope (FEI) operating at 200 kV, and micrographs were recorded with a Gatan Ultrascan CCD camera (2048 × 2048 pixels). For electron tomography, semithick sections were imaged at holder tilt angles between ±60°, with increments of 1°. Data were processed with eTomo and modeled with IMOD 4.9 (University of Colorado, Boulder, [http://bio3d.colorado.edu/imod], as of June 2018). Analytical investigations were carried out with ImageJ (v. 1.51j8) image analysis software, and fill fractions were evaluated from tomogram segments with a standard and automated particle and pattern recognition plug-in (PSA macro for ImageJ, [https://code.google.com/archive/p/psa-macro/], as of June 2018).

### Data availability

The data that support the findings of this study are available from the authors upon reasonable request.

## Electronic supplementary material

Supplementary Information

Peer Review File
