# Supplementary material for: Intertidal exposure favors the soft-studded armor of adaptive mussel coatings
Source: Nat Commun. 2018 Aug 24;9:3424. doi: 10.1038/s41467-018-05952-5 (PMC6109138; doi:10.1038/s41467-018-05952-5)
Supplement: Supplementary file 1 — Supplementary Information [file 41467_2018_5952_MOESM1_ESM.pdf]

Supplementary information

Supplementary figures 1-8

Intertidal exposure favors the soft-studded armor of adaptive mussel coatings

Christophe A. Monnier, Daniel G. DeMartini, J. Herbert Waite

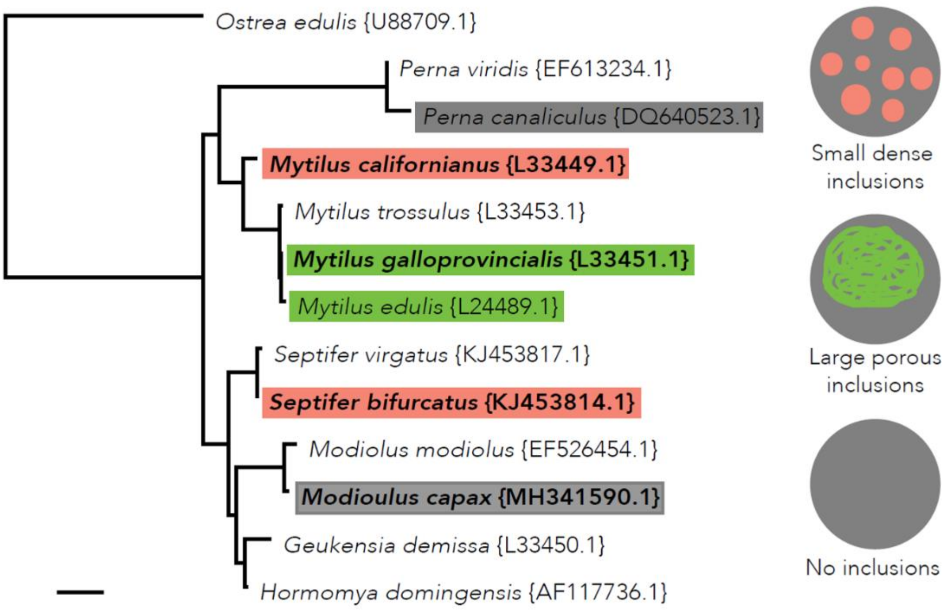

Supplementary Figure 1: Phylogenetic tree showing the general relationships among the family Mytilidae. The species treated in this study are shown in bold (scale bar = 0.01). The tree was generated based on 18S rRNA (accession numbers indicated in brackets) using the Neighbor-joining method, and is in close agreements with other phylogenetic studies of Mytilidae (Distel, 2000, [https://doi.org/10.1006/mpev.1999.0733], as of June 2018). General morphological traits of each cuticle (which have been observed) are indicated by highlighted text, small granular inclusions (red), large porous granular inclusions (green), and no inclusions (gray).

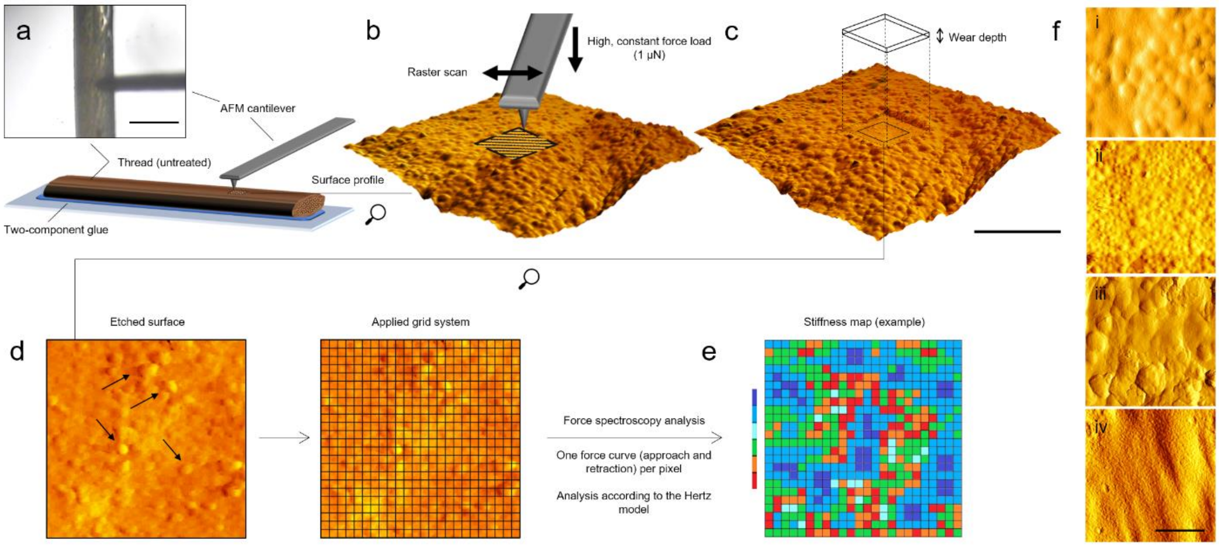

Supplementary Figure 2: Summary of the overall AFM experimental approach. (a) Freshly collected threads (scale bar = 100 µm), mounted on a glass slide with a thin layer of two-component glue, were positioned below the AFM without any further treatment. After imaging the surface in AC mode (b), a high constant force was applied to wear off a specific area in the region of interest (c, scale bar = 2 µm). Subsequent imaging over the same region shows the extent of the damage. Higher resolution images reveal the exposed granular structures within the matrix (e). Representative amplitude channel images of the thread surfaces of all investigated species (i-iv) are shown in f (scale bar = 2 µm).

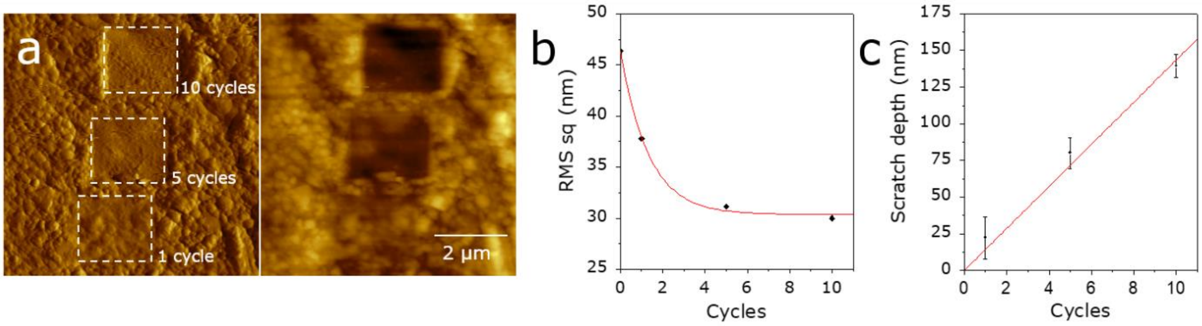

Supplementary Figure 3: Selecting experimental parameters for the scratch tests. (a) Consecutive AFM scratch tests of 1 µN each were conducted on a representative *M. californianus* cuticle as a function of cycle counts. (b) A negative exponential relationship between the amount of scratch cycles and surface roughness is observed (one replicate, 1 x 1 µm), with a plateau being reached after approx. four cycles (*i.e.*, the threshold at which topographic features are overcome). (c) Overall scratch depths (one replicate, 1 x 1 µm, mean ± s.d.) on the other hand exhibit a linear trend with decreasing standard deviations.

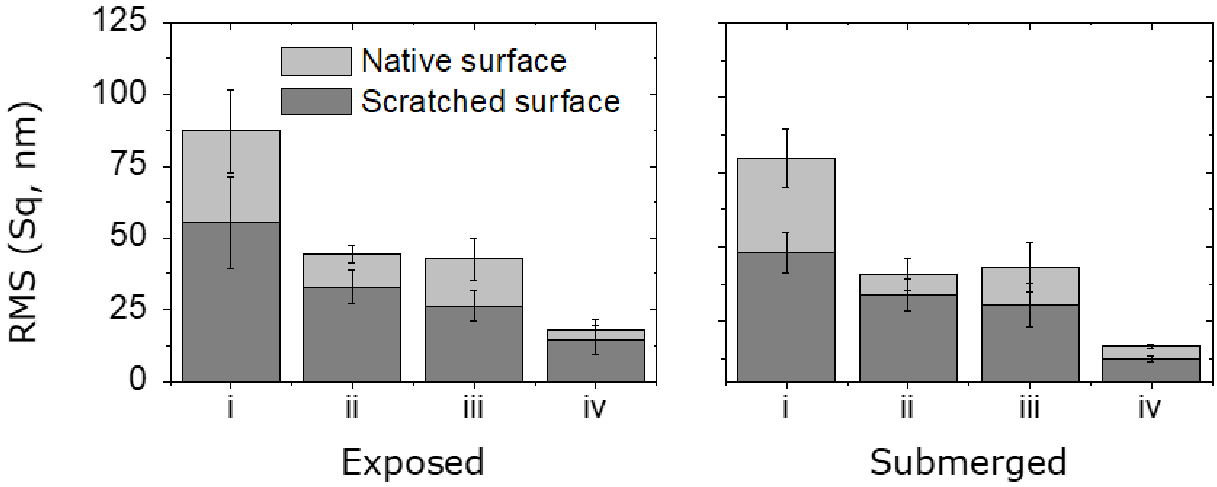

Supplementary Figure 4: Surface roughness values of all investigated cuticles. RMS values of the cuticles of *M. galloprovincialis* (i), *M. californianus* (ii), *S. bifurcatus* (iii) and *M. capax* (iv) prior and after the scratch tests (n = 4 biological replicates, mean ± s.e.m., 1 x 1 µm). Surface roughness does not change between exposed and submerged threads, and is leveled out by the scratch tests.

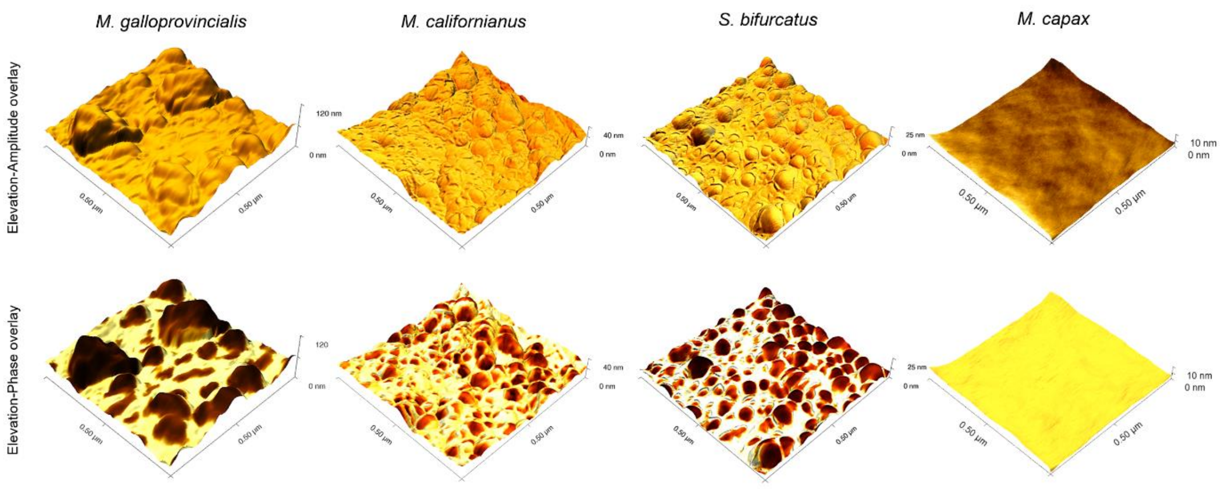

Supplementary Figure 5: AFM AC recordings within the abraded areas. Height profiles overlapped with amplitude (top row) and phase (bottom row) highlight the granular structures, which particularly stand out in the phase channel, in comparison to the granule-free and homogeneous matrix of *M. capax* (right).

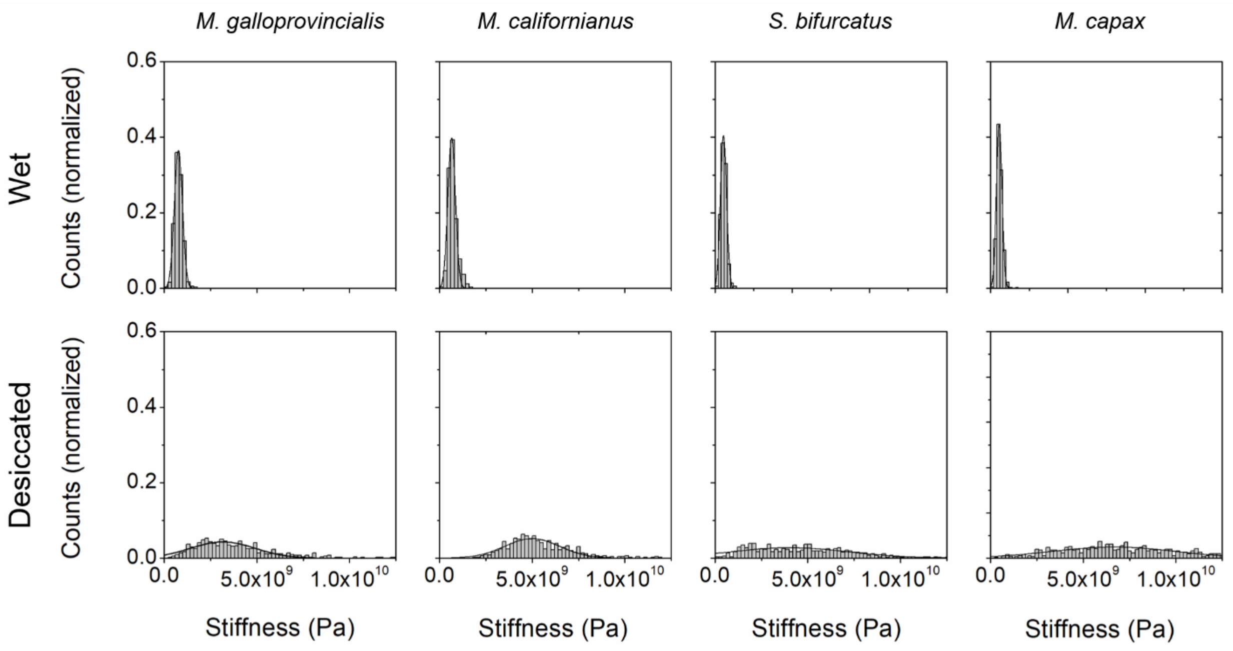

Supplementary Figure 6: Raw force spectroscopy data. Normalized stiffness counts recorded in both fully submerged and desiccated cuticles.

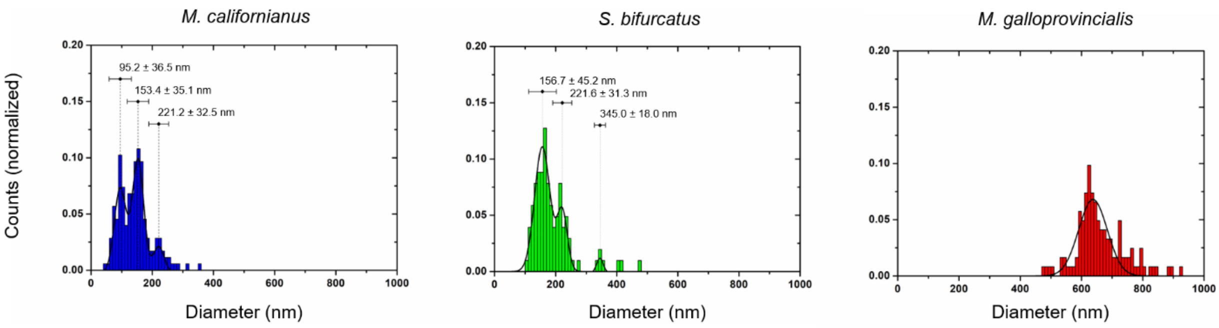

Supplementary Figure 7: Raw size distribution data. Normalized counts obtained from tomograms of the secretory vesicles of all granule-containing mussel species. Peaks were identified by using the Peak Analysis feature of Origin 8.5.

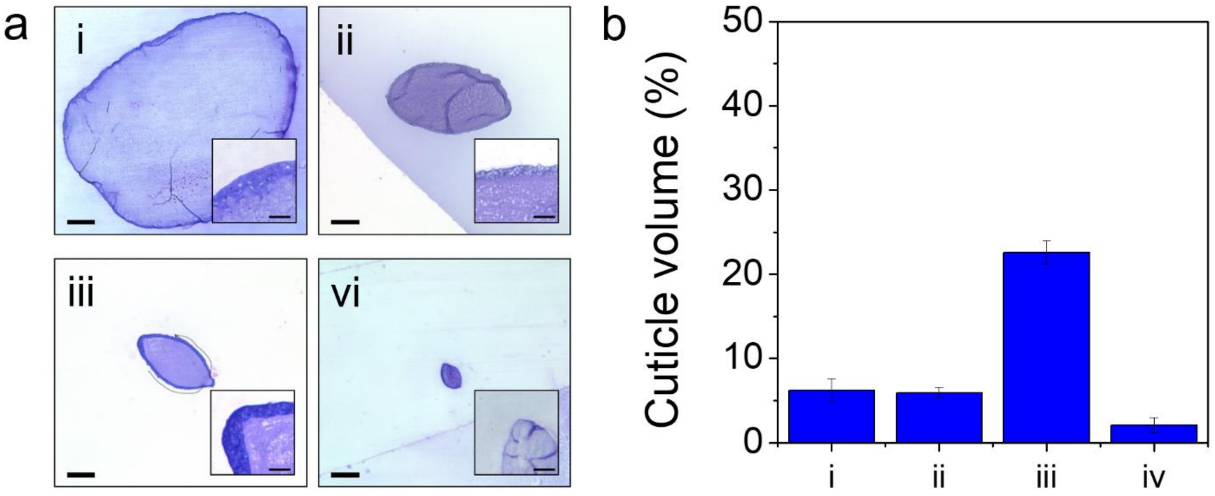

Supplementary Figure 8: The mussel cuticle in relation to the entire thread. (a) Stained lateral cross-sections of the threads of *M. galloprovincialis* (i), *M. californianus* (ii), *S. bifurcatus* (iii) and *M. capax* (iv), viewed by light microscopy (scale bars = 100 µm). The cuticles are especially evident in the insets (scale bars = 5 µm). Cuticle volume fractions in proportion to total thread volume (3 technical replicates, mean ± s.e.m.) are highlighted in (b).
