# Supplementary material for: Intertidal exposure favors the soft-studded armor of adaptive mussel coatings
Source: Nat Commun. 2018 Aug 24;9:3424. doi: 10.1038/s41467-018-05952-5 (PMC6109138; doi:10.1038/s41467-018-05952-5)
Supplement: Supplementary file 2 — Peer Review File [file 41467_2018_5952_MOESM2_ESM.pdf]

Reviewers' Comments:

Reviewer #1:
Remarks to the Author:
The article by Monnier et al describes the morphology and nano-mechanical properties of the protecting layer cuticle of the byssal threads of marine mussels of 4 species. They use electron microscopy and tomography, scratch tests in the AFM and force spectroscopy. Contrary to previous suggestions, they show that the granules within the cuticle are threefold softer than the surrounding matrix at intermediate hydration levels. When fully hydrated there is no difference between the mechanical properties of the granules and the matrix where they are both softer than partially hydrated. Whereas in fully desiccated state, the stiffness values are similar between matrix and granules but are higher than in the hydrated state. The authors suggest that the granules are more hygroscopic. The authors correlate these observations with the habitat of the different species.
Overall the work is interesting important. I have a major concern related to the scratch test mechanical analysis. Indeed, of the mechanical tests presented in the manuscript, I tend to believe the force spectroscopy more than the scratch test. This is because of the known effect of topography on the scratch tests results. It would be important to address this issue. For example, scratch tests on cross-sections of the threads could test the abrasion hypothesis.
minor comments:
1) In all the figures, the text and numbers are too small
2) It would be useful to present an overlay of Fig. 3b and c at least in supporting information.
3) In figure 2 n=4 refers to biological or technical replicates?

Reviewer #2:
Remarks to the Author:
This study offers an in-depth investigation of the micro- and nano- structure and mechanics of the cuticle of mussel byssal threads. It follows on prior work from the lab group, which concluded the renowned ‘iron-clad’ toughness of the cuticle stemmed from the combination of stiff granules embedded in a soft matrix. Using AFM scratch tests, the authors conclusively reverse this view, demonstrating the granules are instead much softer than the surrounding matrix. This aspect of the work is convincingly presented (e.g., Fig. 3) and, because mussel byssus has served as the bio- inspiration for the design of tough and durable engineered materials, is of broad interest.
The TEM images of the secretory vesicles and the cuticles are stunning, and Fig. 1 is thoughtfully laid out. The comparison among species is a nice addition to this study; it is surprising how much variation there is in the size and structure of the secretory vesicles and the cuticle they create.
I have two major problems with the manuscript. The first is the rationale for the work is poorly developed. On page 2, the authors state there are ‘many questions unanswered’ but they are not sufficiently spelled out. Why would one care about de-/rehydration cycles? Who says granular morphologies are ‘distinctly unique and adaptive’ and if they are adaptive, doesn’t that imply the ‘functional advantages’ are already known? As written, the study is too exploratory and seems more driven by available technology than addressing any specific hypotheses.
Second, the interpretation of the ‘adaptive’ function of the cuticles needs to be toned down considerably. The hygroscopy of the cuticles was not measured, nor was their ability to retard desiccation. Is desiccation even an issue for the byssal threads of intertidal mussels? The animals trap water in their shells so presumably the local relative humidity is quite high; many desiccation intolerant critters seek refuge in mussel aggregations. Moreover, there is no consideration of the phylogenetic relationships among the species. Much is made of the differences in the species’ distribution with respect to tidal height and how they might relate to cuticle properties, but the observed variation could just be a case of comparing apples to oranges to bananas without sufficient replication. Overall, there is just too much conjecture about function and adaptation that outpace the data that are presented.

Reviewer #3:
Remarks to the Author:
The article by Monnier et al. presents new mechanical characterisation of the mussel's cuticle, more specifically the role of each phase in this natural particulate composite. The cuticle is composed of granule dispersed within an amorphous matrix, with an amount of this granule changing from species to species. The report begins with a connection between the habitat of the different species with the quantity of granules, the more subject to stress from tides and emersion time is the cuticle, the more granules are present. This connection can be explained via two hypotheses: either the granules act as mechanical reinforcement and prevent the degradation of the cuticle, or they act as water reservoir and prevent the drying of the thread as such drying would result in an increased brittleness. The latter is one the authors try to support through small scale mechanical probing and imaging.
The results the authors assembled are compelling, starting from the amount of granules increasing with time spent out of water. The resistance to a scratch test shows that a higher number of granules translates into a higher degradation. The lower mechanical properties of the granules are confirmed by indentation performed with the AFM tip and shows stiffness several times lower than that of the amorphous phase. The detrimental effect of drying is also measured and is represented by a higher global stiffness of the samples and a larger spread of the local values. Finally, electron tomography results are presented and linked to the formation of the cuticle.
I find the article quite interesting and while its conclusion seems to go against prior knowledge, it seems to be the first time that mechanical properties of the cuticle are probed at these length scales and the amount of concordant results makes it convincing in my opinion. These findings could help the design of coatings that are subjected to the same rough conditions.

However, I have some questions and remarks for the authors to clarify the methods used and results:
- Given the audience of this journal, the authors should add some information on the type of information extracted by the scratch tests and force maps. The fact that the phase images are more sensitive to the materials stiffness than the topography is quite important to understand the results. The hertzian contact model used for the Young modulus calculation should be explicitly provided in order to make the results reproducible by others.
- Why only the already abraded surfaces were characterised by force spectroscopy? It seems to me that the analysis could have been done on fresh surface without risking the surface properties being altered by the scratching.
- Are the fill fraction measured volume fraction? It is unclear from the text if they were measured as surface coverage or not. The plugins used for the measurement should be mentioned and if a paper describing them exists it should be cited to provide the exact methodology to the reader and ensure reproducibility of the method.

On a more aesthetic perspective:

- Why the stiffness scales are all inverted (the higher values at the bottom)?
- The x axis of all the histogram plots are wrong (either the results are in Pa with the 10^9 or in GPa without) and with a really small font size
- About the title, and it is only a suggestion as this is more a matter of taste, but while the use of idioms can be enticing, it is very puzzling for non-native speaker and makes the topic of the paper rather obscure.

Response to reviewers' comments

Reviewer 1:

The article by Monnier et al describes the morphology and nano-mechanical properties of the protecting layer cuticle of the byssal threads of marine mussels of 4 species. They use electron microscopy and tomography, scratch tests in the AFM and force spectroscopy. Contrary to previous suggestions, they show that the granules within the cuticle are threefold softer than the surrounding matrix at intermediate hydration levels. When fully hydrated there is no difference between the mechanical properties of the granules and the matrix where they are both softer than partially hydrated. Whereas in fully desiccated state, the stiffness values are similar between matrix and granules but are higher than in the hydrated state. The authors suggest that the granules are more hygroscopic. The authors correlate these observations with the habitat of the different species.

- Overall the work is interesting important. I have a major concern related to the scratch test mechanical analysis. Indeed, of the mechanical tests presented in the manuscript, I tend to believe the force spectroscopy more than the scratch test. This is because of the known effect of topography on the scratch tests results. It would be important to address this issue. For example, scratch tests on cross-sections of the threads could test the abrasion hypothesis.

We have added additional information and experiments to the SI to clarify the thinking behind our dependence on both scratch and indentation tests. Indeed, topographic roughness can have an influence, and we have enhanced this aspect in the respective section. For these measurements however, we set our experimental parameters (in particular the applied force and cycle number) to significantly outweigh these effects. Moreover, we observed that while the topography between exposed and submerged states does not change, the wear depth does, which is an indication that mechanical properties are primarily at play here. To highlight this, we added all RMS sq values to the SI, reformulated the section in question and have added these queries in detail. The section has been rephrased as such:

"As seen in AFM images recorded within the abraded areas, multiple granular structures, which are otherwise covered by matrix material (Figure 1d), are clearly distinguishable (especially in *M. californianus*, Figure 3a, Suppl. Figure 2d) and apparently intact (Suppl. Figure 5). More comprehensive investigations were thus carried out on these uncovered granules despite possible alterations of their properties through the scratch tests. In doing so however, potential artifacts arising from microscale sectioning treatments (*e.g.*, embedding, chemical fixation, freezing or slicing), which are even more likely to perturb overall structure and biomechanics than scratch tests, were avoided."

While we did attempt these experiments on cross-sections, these surfaces were not necessarily flatter than the cuticle surface (i.e., presumably because of unequal swelling/contraction of the different cuticle phases during the cutting). Embedding and sectioning the sample would have avoided this issue, but substantially altered the mechanical properties at the same time. We thus opted to preserve the native conditions to a maximum degree instead.

- Minor comments:

1) In all the figures, the text and numbers are too small

We thank the reviewer for pointing this out. We have changed the fonts of all numbers and text in the figures accordingly.

2) It would be useful to present an overlay of Fig. 3b and c at least in supporting information.

Attempts were made to overlay these two datasets. However, the resulting composites unfortunately did not look very compelling, mainly due to the resolution mismatch between both images. As an alternative, we changed the layout of Figure 3 to put them in a more relatable context to one another.

3) In figure 2 n=4 refers to biological or technical replicates?

These values from the scratch tests are biological replicates, whereas the tomographic ones are technical. We have updated the figure legend accordingly.

Reviewer 2:

This study offers an in-depth investigation of the micro- and nano- structure and mechanics of the cuticle of mussel byssal threads. It follows on prior work from the group, which concluded that the renowned 'iron-clad' toughness of the cuticle stemmed from the combination of stiff granules embedded in a soft matrix. Using AFM scratch tests, the authors conclusively reverse this view, demonstrating the granules are instead much softer than the surrounding matrix. This aspect of the work is convincingly presented (e.g., Fig. 3) and, because mussel byssus has served as the bio- inspiration for the design of tough and durable engineered materials, is of broad interest.

The TEM images of the secretory vesicles and the cuticles are stunning, and Fig. 1 is thoughtfully laid out. The comparison among species is a nice addition to this study; it is surprising how much variation there is in the size and structure of the secretory vesicles and the cuticle they create.

- I have two major problems with the manuscript. The first is the rationale for the work is poorly developed. On page 2, the authors state there are 'many questions unanswered' but they are not sufficiently spelled out. Why would one care about de-/rehydration cycles? Who says granular morphologies are 'distinctly unique and adaptive' and if they are adaptive, doesn't that imply the 'functional advantages' are already known? As written, the study is too exploratory and seems more driven by available technology than addressing any specific hypotheses.

We appreciate this assessment and have accordingly modified the manuscript. This includes a major reformulation of the introduction and parts of the results to state more clearly what we intended to investigate. The section has been rephrased as such:

"To better understand the material properties of these coatings and to explore the correlation between architecture and wear, we examined the cuticles of four local mussel species with different granular morphologies. *In situ* atomic force microscopy (AFM) was used to identify the scratch-resistant and mechanical features at a nanometer resolution, and complemented with both transmission electron microscopy (TEM) images and tomograms of the cuticles and their precursors."

- Second, the interpretation of the 'adaptive' function of the cuticles needs to be toned down considerably. The hygroscopy of the cuticles was not measured, nor was their ability to retard desiccation. Is desiccation even an issue for the byssal threads of intertidal mussels? The animals trap water in their shells so presumably the local relative humidity is quite high; many desiccation intolerant critters seek refuge in mussel aggregations. Moreover, there is no consideration of the phylogenetic relationships among the species. Much is made of the differences in the species' distribution with respect to tidal height and how they might relate to cuticle properties, but the observed variation could just be a case of comparing apples to oranges to bananas without sufficient replication. Overall, there is just too much conjecture about function and adaptation that outpace the data that are presented.

We have considerably toned down the adaptive function of the cuticle and have added a phylogenetic tree to the supporting information file. However, we do stand by the claim that desiccation is a major issue for the performance of these materials. Rocks and their attached threads heat up substantially in the sunlight and are also exposed to high winds during tidal exposure. Although mussel interiors are shielded from these effects, the distal thread portions often are not. The embrittlement of even a few thread cuticles during each intertidal exposure could add additional costs to the already substantial energy budget of byssus. Given that we observed a clear a mechanical deterioration in the nanomechanical tests, and that this feature is only a threat to intertidal species, we still believe that relevant environmental boundary conditions have been explored. We maintain that the number of correlated methods and results are strongly indicative that we are not comparing apples to bananas (or rather that we are comparing valid features between them).

Reviewer 3:

The article by Monnier et al. presents new mechanical characterisation of the mussel's cuticle, more specifically the role of each phase in this natural particulate composite. The cuticle is composed of granule dispersed within an amorphous matrix, with an amount of this granule changing from species to species. The report begins with a connection between the habitat of the different species with the quantity of granules, the more subject to stress from tides and emersion time is the cuticle, the more granules are present. This connection can be explained via two hypotheses: either the granules act as mechanical reinforcement and prevent the degradation of the cuticle, or they act as water reservoir and prevent the drying of the thread as such drying would result in an increased brittleness. The latter is one the authors try to support through small scale mechanical probing and imaging.

The results the authors assembled are compelling, starting from the amount of granules

increasing with time spent out of water. The resistance to a scratch test shows that a higher number of granules translates into a higher degradation. The lower mechanical properties of the granules are confirmed by indentation performed with the AFM tip and shows stiffness several times lower than that of the amorphous phase. The detrimental effect of drying is also measured and is represented by a higher global stiffness of the samples and a larger spread of the local values. Finally, electron tomography results are presented and linked to the formation of the cuticle.

I find the article quite interesting and while its conclusion seems to go against prior knowledge, it seems to be the first time that mechanical properties of the cuticle are probed at these length scales and the amount of concordant results makes it convincing in my opinion. These findings could help the design of coatings that are subjected to the same rough conditions.

However, I have some questions and remarks for the authors to clarify the methods used and results:

- Given the audience of this journal, the authors should add some information on the type of information extracted by the scratch tests and force maps. The fact that the phase images are more sensitive to the materials stiffness than the topography is quite important to understand the results. The hertzian contact model used for the Young modulus calculation should be explicitly provided in order to make the results reproducible by others.

We and have extended the section in question, and included an additional reference. The specifics for the Hertzian model calculation were added as well to the materials and methods section:

"AFM phase imaging, which takes advantage of viscoelastic differences to generate contrast over topographic features[28] highlights the granules particularly well at these length scales (Figure 3b, Suppl. Figure 5)."

"Stiffness values were extracted with the Asylum Research MFP-3D Hertz analysis tool by using the upper 60 % of the approach curve, a half-angle of 20° and a Poisson ratio of 0.33 to fit the model."

- Why only the already abraded surfaces were characterised by force spectroscopy? It seems to me that the analysis could have been done on fresh surface without risking the surface properties being altered by the scratching.

This method was chosen over other approaches to remove/avoid measuring components other than the cuticle. Given that the surface composition is unknown (eg. whether there is a thin, invisible layer on top or whether it is plain) and likely contaminated with external debris, we wanted to avoid any external disturbance of the mechanical measurements. Moreover, the outer cuticular surface of at least one type of byssal thread is consistently covered by a thin layer of

polysaccharides. Besides, effective measurements of the granules from the surface would have been skewed by the thin (20-40nm) layer of matrix material covering them. Microtoming was considered as well, but this procedure would have arguably altered the mechanical properties to an even greater extent.

- Are the fill fraction measured volume fraction? It is unclear from the text if they were measured as surface coverage or not. The plugins used for the measurement should be mentioned and if a paper describing them exists it should be cited to provide the exact methodology to the reader and ensure reproducibility of the method.

The presented values are volume fractions, and we have updated the sections accordingly. We have also added the link and explanation to the ImageJ plugin to the materials and methods section.

"Analytical investigations were carried out with ImageJ (v. 1.51j8) image analysis software, and fill fractions were evaluated from tomogram segments with a standard and automated particle and pattern recognition plug-in (PSA macro for ImageJ, https://code.google.com/archive/p/psa-macro/, as of May 2018)."

On a more aesthetic perspective:

- Why the stiffness scales are all inverted (the higher values at the bottom)?

We have inverted all color bars.

- The x axis of all the histogram plots are wrong (either the results are in Pa with the 10^9 or in GPa without) and with a really small font size

We thank the reviewer for pointing this out and have corrected the labels, and have increased all font sizes.

- About the title, and it is only a suggestion as this is more a matter of taste, but while the use of idioms can be enticing, it is very puzzling for non-native speaker and makes the topic of the paper rather obscure.

We are happy to comply with another title if the editor agrees with this assessment. A parallel suggestion without idioms:

Adaptive mussel coatings: intertidal exposure favors soft-studded armors

We wish to thank the reviewers for their comments and suggestions and hope that we could address their concerns accordingly.

Reviewers' Comments:

Reviewer #1:
Remarks to the Author:
The authors have answered all my concerns. In my view the article may be published in its present form.

Reviewer #2:
Remarks to the Author:
I have reviewed the authors responses to my previous comments and find they have all been satisfactorily addressed. The rationale is each experiment is now clearly established in the introduction and the flow of the entire manuscript is greatly improved.

Reviewer #3:
Remarks to the Author:
The authors answered to my questions and comments thoroughly. I recommend this article for publication.
